# Monitoring the 5′UTR landscape reveals isoform switches to drive translational efficiencies in cancer

**DOI:** 10.1038/s41388-022-02578-2

**Published:** 2022-12-23

**Authors:** Ramona Weber, Umesh Ghoshdastider, Daniel Spies, Clara Duré, Fabiola Valdivia-Francia, Merima Forny, Mark Ormiston, Peter F. Renz, David Taborsky, Merve Yigit, Martino Bernasconi, Homare Yamahachi, Ataman Sendoel

**Affiliations:** 1 Institute for Regenerative Medicine (IREM), University of Zurich, Wagistrasse 12, CH-8952 Schlieren-Zurich, Switzerland; 2 Life Science Zurich Graduate School, Molecular Life Science Program, University of Zurich/ETH Zurich, Zurich, Switzerland

**Keywords:** Cancer genomics, Nutrient signalling

## Abstract

Transcriptional and translational control are key determinants of gene expression, however, to what extent these two processes can be collectively coordinated is still poorly understood. Here, we use Nanopore long-read sequencing and cap analysis of gene expression (CAGE-seq) to document the landscape of 5′ and 3′ untranslated region (UTR) isoforms and transcription start sites of epidermal stem cells, wild-type keratinocytes and squamous cell carcinomas. Focusing on squamous cell carcinomas, we show that a small cohort of genes with alternative 5′UTR isoforms exhibit overall increased translational efficiencies and are enriched in ribosomal proteins and splicing factors. By combining polysome fractionations and CAGE-seq, we further characterize two of these UTR isoform genes with identical coding sequences and demonstrate that the underlying transcription start site heterogeneity frequently results in 5′ terminal oligopyrimidine (TOP) and pyrimidine-rich translational element (PRTE) motif switches to drive mTORC1-dependent translation of the mRNA. Genome-wide, we show that highly translated squamous cell carcinoma transcripts switch towards increased use of 5′TOP and PRTE motifs, have generally shorter 5′UTRs and expose decreased RNA secondary structures. Notably, we found that the two 5′TOP motif-containing, but not the TOP-less, *RPL21* transcript isoforms strongly correlated with overall survival in human head and neck squamous cell carcinoma patients. Our findings warrant isoform-specific analyses in human cancer datasets and suggest that switching between 5′UTR isoforms is an elegant and simple way to alter protein synthesis rates, set their sensitivity to the mTORC1-dependent nutrient-sensing pathway and direct the translational potential of an mRNA by the precise 5′UTR sequence.

## Introduction

Gene expression is tightly regulated in space and time to determine cellular function and behavior. Transcriptional and translational control are key steps in the gene expression pathway; however, to what extent these two processes can be collectively coordinated is still poorly understood. Recently, several studies have suggested a non-canonical mode of regulation by which a large cohort of yeast genes switch between short and long 5′ untranslated regions (UTRs) while keeping the coding sequences (CDSes) identical [1–4]. Since 5′UTRs are critical for ribosome recruitment and ultimately initiation of translation [5], switching between these 5′UTR isoforms resulted in differential translational efficiencies of these mRNAs. During yeast meiosis, for instance, around 8% of genes were temporally regulated by an extended 5′UTR that was poorly translated due to the presence of inhibitory upstream open reading frames (uORFs) [2]. These examples highlight the possibility that the presence or absence of regulatory sequences set by the exact 5′UTR isoform could directly control protein synthesis rates of an mRNA and justify the need to accurately determine the precise transcription start sites (TSS) and 5′UTR isoforms.

The 5′UTR sequence also plays a critical role in the mammalian target of rapamycin complex 1 (mTORC1) nutrient-sensing pathway, which selectively enhances the translation rates of 5’terminal oligopyrimidine (TOP) and pyrimidine-rich translational element (PRTE) motif-containing mRNAs [6, 7]. mTORC1 signaling is commonly activated in tumorigenesis and deregulated mTORC1 signaling is implicated in cancer progression [8]. Among the mTORC1-driven cellular processes, mTORC1-dependent translational reprogramming is likely among the most critical [6, 9], highlighting the importance of understanding the repertoire of mRNAs that mediate mTORC1-dependent cancer progression.

In this study, we used Nanopore long-read sequencing and cap analysis of gene expression (CAGE-seq) to document the UTR isoform landscape of epidermal stem cells in vivo, wild-type keratinocytes and cultured squamous cell carcinoma cells (SCC^c^). We provide the combined dataset and de novo transcriptome annotations as a resource on an easily accessible genome browser (link provided in Material & Methods). Moreover, by combining polysome profiling with CAGE-seq, we determine the translational efficiency for each 5′ transcript isoform and demonstrate that highly translated squamous cell carcinoma transcripts show increased use of 5′UTR motifs that are known to drive mTORC1-dependent translation of the mRNA. Notably, we found that the two TOP motif-containing, but not the TOP-less, *RPL21* transcript isoforms strongly stratified overall survival in human head and neck squamous cell carcinoma patients and resulted in medium overall survival differences of up to 3.5 years.

Our findings demonstrate the complexity of TSS selection in the mammalian genome and suggest that even slight differences in the precise TSS and the resulting 5′UTR isoform can vastly alter the translational efficiencies of an mRNA. Given the heterogeneity of TSSes for genes with broad promoters, our data also caution against solely relying on the annotated transcripts. Although previously appreciated for tissue-specific TOP mRNAs [10], switching between TOP/PRTE and non-TOP/PRTE motif-containing 5′UTR isoforms is an elegant and simple way to effectively alter translational efficiencies of a cohort of translation-associated genes in cancer. More generally, since alternative transcription start sites [11] and alternative splicing events are widespread [12], the set of post-transcriptional regulatory elements such as RNA-binding protein (RBP) binding sites, uORFs [2, 3], or 5′TOP motifs could be widely used in cancer to link transcription and translation and direct the translational potential of an mRNA by the precise 5′ transcript sequence.

## Results

**Fig. 1:**
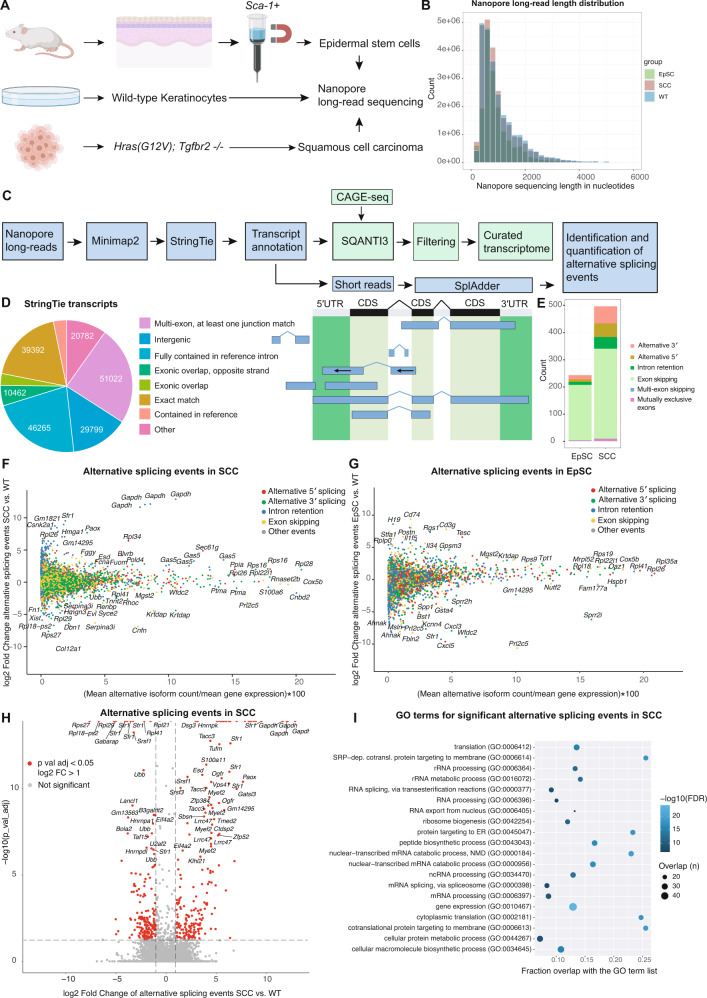
Nanopore long-read sequencing identifies alternative mRNA isoforms in the mouse skin. **A** Experimental outline for the isolation of SCA-1+ epidermal stem cells (EpSC), wild-type keratinocytes (WT) and cultured squamous cell carcinoma cells (SCC^c^) used for Nanopore long-read sequencing. **B** Read length distribution for the Nanopore long-read sequencing data set of epidermal stem cells (EpSC), wild-type keratinocytes (WT), and squamous cell carcinomas (SCC^c^). The mean read length was between 943-1035 bp (EpSC: 943 bp, WT: 1035 bp, SCC^c^: 994 bp). **C** Bioinformatic pipeline for the processing of the Nanopore long-read sequencing data to identify and quantify alternative isoforms in epidermal stem cells and squamous cell carcinoma cells. Nanopore long-reads were mapped and a StringTie transcriptome was built. Transcripts were then filtered by the SQANTI3 pipeline and a quality control report was created (M&Ms). As an additional filtering step, transcripts were confirmed by CAGE-seq transcription start sites (TSSes) to build a curated transcriptome. Based on the transcriptome annotation and the short-read RNA sequencing data, SplAdder was used for the identification and quantification of alternative splicing events. **D** Quantification and categorization of the total transcript numbers identified by StringTie using the Nanopore long-read sequencing data of SCA-1+ epidermal stem cells (EpSCs), wild-type keratinocytes, and squamous cell carcinomas (SCC^c^) before filtering by SQANTI3 and CAGE-seq. Right panel shows examples for the different categories as defined by StringTie for transcript identifications, and the left panel shows the respective numbers. **E** Numbers and categories of significantly changed splicing events in epidermal stem cells (EpSC) and squamous cell carcinomas (SCC^c^), compared to wild-type keratinocytes, as identified by the SplAdder pipeline. **F**, **G** The landscape of alternative isoforms in squamous cell carcinoma cells or epidermal stem cells compared to wild-type keratinocytes using SplAdder, which quantifies and tests alternative splicing events. Color-coded are alternative 5′ splicing, 3′ splicing, intron retention and exon skipping events. The x-axis shows the alternatively spliced isoforms as a percentage of total gene expression, the y-axis shows the fold change of the alternative event. **H** Volcano plot showing the significant alternative splicing events and fold changes of splicing events in squamous cell carcinoma cells (SCC^c^) compared to wild-type keratinocytes (WT). Red indicates a significant >2× fold change in alternative splicing events. **I** Gene expression (GO:0010467) is the top gene ontology (GO) term for alternatively spliced genes in squamous cell carcinomas (SCC^c^) and is mainly driven by ribosomal proteins and splicing factors. GO term analysis shows the top 20 GO term hits (alphabetically ordered) with their false discovery rate (FDR, blue tone) and overlap with the GO term gene list in numbers (size of circle) and the fraction (x-axis).

To systematically monitor the landscape of the full-length transcriptome, we performed Nanopore long-read RNA sequencing of in vivo mouse epidermal stem cells and cultured squamous cell carcinoma cells (SCC^c^) to exemplify distinct biological contexts of the mouse skin. First, we isolated interfollicular epidermal stem cells (EpSCs) of P60 (postnatal day 60) adult mice by a rapid magnetic-activated cell sorting protocol using anti-stem cell antigen-1 microbeads (*Sca-1*, encoded by the *Ly6a* gene) [13, 14]. SCC^c^ were obtained from a previously established tumor allograft model, driven by oncogenic *Hras*^*G12V*^ in combination with loss of the TGFβ receptor II, which rapidly form invasive squamous cell carcinomas when injected into immunocompromised mice [15]. As a reference, we used wild-type keratinocytes (Fig. 1A).

In total, we generated 158.1 million reads with a mean read length between 943–1035 bp (Fig. 1B). For transcriptome de novo assembly, we first used StringTie [16], which allows accurate reconstructions and quantitation of genes and transcripts. Comparing the long-read sequences to the reference mouse genome, we identified a total of 210′630 transcripts, including 39′392 transcripts with an exact match of the intron chain to the reference genome (Fig. 1C, D). For ~62% of genes, we detected only one transcript, while another ~17% of genes contained either two or three transcripts, and the remaining ~21% had four or more transcripts (Supplementary Fig. 1A). In addition, to classify and characterize the long-read transcript sequences, we used SQANTI3, which included further filtering resulting in 149,434 transcripts (Supplementary Fig. 1B, QC report in M&Ms). Finally, we filtered out transcripts not supported by our corresponding CAGE-seq dataset, resulting in a curated annotation with 77′017 transcripts (Fig. 1C, Supplementary Fig. 1B, QC reports in M&Ms).

**Fig. 2:**
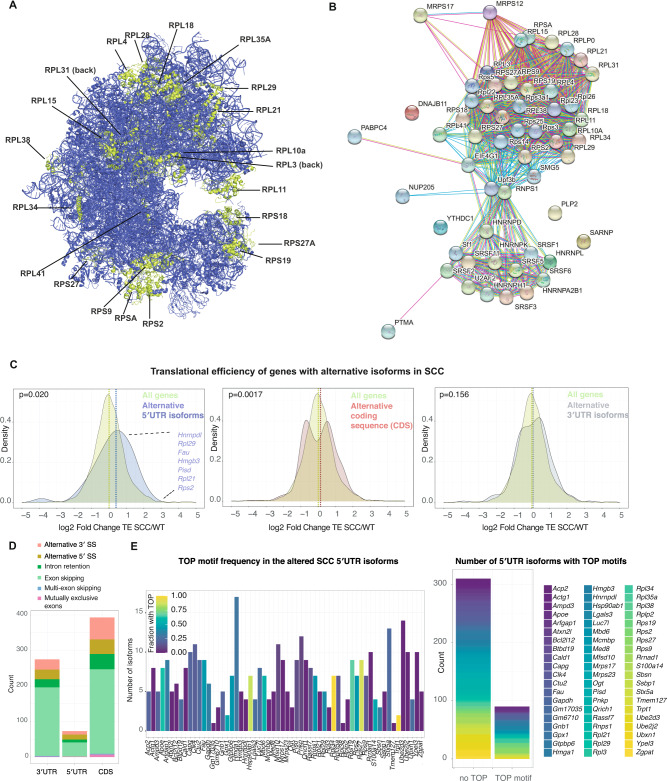
Genes with alternative 5′UTR isoforms show increased translational efficiencies in squamous cell carcinomas. **A** Localization of ribosomal proteins on the human 80S ribosome with significantly changed alternative splicing events of their encoding mRNAs in squamous cell carcinomas. Note that *RPLP0* and *RPLP2* were not included in the structure. **B** STRING interaction network analysis for the alternatively spliced genes in the GO term gene expression (GO:0010467) in squamous cell carcinomas compared to wild-type keratinocytes. **C** The translational efficiency of genes with differential alternative splicing events in the 5′UTR is increased in squamous cell carcinomas (SCC^c^). Fold change in translation efficiency (TE) was computed for all genes or genes with significant alternative splicing events in the 5′UTR, coding sequence (CDS) or 3′UTR. Translation efficiency (ribosome profiling reads divided by RNA-seq reads) was computed using the LRT-test of the DESeq2 package. *P*-values indicate a two-sample Kolmogorov–Smirnov test comparing the TE distribution of genes with alternative splicing events to all genes. **D** Numbers and splicing categories of significantly changed alternative splicing events in squamous cell carcinomas (SCC^c^) that alter either the 5′UTR, the coding sequence (CDS) or the 3′UTR. SS splice sites. **E** Most genes with differential 5′UTR isoforms in squamous cell carcinomas (SCC^c^) express a set of transcripts that contain or exclude TOP motifs. StringTie transcripts of the 5′UTR isoforms and their 5′UTR sequences were assessed for the presence of TOP motifs as defined by a C at the +1 position and an unbroken series of 4–16 pyrimidines. Left panel shows the different 5′UTR isoform genes, while the colors indicate the fraction of StringTie transcripts that contain a 5′TOP motif between 0 and 1 (0 and 100%). Right panel, number of isoforms within the cohort of genes with 5′UTR isoforms in SCC^c^ that either contains a 5′TOP or do not contain a TOP motif. The color refers to the genes within the cohort of 5′UTR isoforms defined on the right side.

To compute the occurrence of alternative isoforms and their differential expression in EpSCs and SCC^c^ versus wild-type keratinocytes, we exploited the SplAdder toolbox [17] (Fig. 1E–H). Using maximally stringent SplAdder confidence parameters, we found a total of 496 and 243 significantly changed isoform events in squamous cell carcinomas and epidermal stem cells, respectively. The identified alternative isoform events mainly fall into the categories of 5′ and 3′ alternative splice sites, exon skipping, and intron retention (Fig. 1E, Supplementary Fig. 1A, B). While in EpSCs, exon skipping events were prevailing, SCC^c^ additionally exhibited 50 alternative 5′ and 63 alternative 3′ splicing events (Fig. 1E). The alternative splicing events were observed across a wide range of relative isoform abundances compared to the corresponding gene expression in SCC^c^ and EpSCs (Fig. 1F, G). Genes with significant alternative splicing events in SCC^c^ were enriched in ribosomal proteins and splicing factors (Fig. 1H, I) and included a total of 23 ribosomal protein genes of the 40S and 60S ribosomal subunits (Fig. 2A, B, Supplementary Fig. 1C, D). Of note, many of these ribosomal proteins are located on the surface of the ribosome (Fig. 2A, B) and include for instance *Rpl38*, previously implicated in the preferential translation of specific subpools of mRNA [18]. We also found that the 5′UTRs of these 23 differentially spliced ribosomal proteins exhibited higher GC content and were more structured than the rest of the ribosomal proteins (Supplementary Fig. 1I). Furthermore, the total number of detected ribosomal protein mRNA isoforms increased from 264 in WT to 290 in SCC^c^. Together, our analyses document the landscape of alternative transcript isoforms and suggest that in squamous cell carcinomas, a small cohort of translation-related genes is differentially spliced.

Given that the 5′UTR is a critical determinant of mRNA translation rates [5], we next focused on the alternative 5′UTR isoforms in SCC^c^ and asked how these 5′UTR isoforms affect translational efficiencies (TE). To determine which regions of the transcripts are affected by the alternative isoform usage, we further sub-grouped the significant alternative splicing events in SCC^c^ and found that 73 and 275 events altered the 5′UTR and 3′UTR sequences, respectively. Out of the 23 differentially spliced ribosomal proteins, 12 contained alternative 5′UTR isoforms. The identified alternative isoform events were mainly resulting from 5′ and 3′ alternative splice sites, intron retention, and exon skipping (Fig. 2D). We then exploited a previously published ribosome profiling and short-read RNA-seq data set carried out in the identical wild-type keratinocyte and *Hras*^*G12V*^*; Tgfbr2 null* squamous cell carcinoma lines to compute differential translational efficiencies as a measure of protein synthesis rates per mRNA molecule [19]. Of note, translational efficiency was assessed at the gene level, with the caveat that low abundant isoforms with altered TEs would not be detected in overall TE changes. When the differential isoform usage between SCC^c^ and wild-type keratinocytes occurred in the 3′UTR, overall translational efficiencies of the altered genes were unaffected and exhibited a similar overall TE distribution compared to all genes (Fig. 2C, D). In contrast, we found that the genes that exhibited significant alternative 5′UTR events resulted in a significant shift with an overall increase in translational efficiencies (Fig. 2C, median log2 fold change 0.23 vs. 0.011 in 5′UTR isoform genes vs. all genes). Furthermore, the cohort of genes with alternative events in the CDS also showed a significant shift, however, with a bimodal distribution that only slightly altered the median translational efficiency (Fig. 2C, D, median log2 fold change 0.13 vs. 0.011 in CDS isoform genes vs. all genes). These data suggest that squamous cell carcinomas differentially express alternative 5′UTR isoforms of a small cohort of genes to overall increase their protein synthesis rates.

mRNAs that encode translation factors typically possess 5′ terminal oligopyrimidine (TOP) motifs, which are essential for the coordinated translation of the family of TOP mRNAs [10]. The translation of TOP motif-containing mRNAs is orchestrated by the mammalian target of rapamycin complex 1 (mTORC1) nutrient-sensing signaling pathway [6, 7]. TOP motifs are defined as a + 1 cytidine (C) directly adjacent to the 5′ cap structure, followed by an unbroken series of 4–16 pyrimidines. To determine the number of TOP motifs in the alternative 5′UTR isoforms of SCC^c^, we extracted the de novo 5′ StringTie transcriptome sequences of the genes with altered 5′UTR isoforms and analyzed them for the occurrence of TOP motifs. We found that most genes expressing altered 5′UTR isoforms in SCC^c^ contain both non-TOP and TOP motifs (Fig. 2E), indicating the possibility of a cell to toggle between mTORC1-dependent and mTORC1-independent translation.

To test this possibility further, we next focused on 5′TOP occurrence. A previous study had found that many 5′TOP motifs are – rather than a binary decision – part of a broader continuum of transcription start sites (TSS), whose combination of TOP and non-TOP motif transcripts within a TSS window specify the overall strength of mTORC1-dependent translational regulation of a 5′UTR [10]. To accurately predict the regulation by mTORC1/LARP1, they introduced a so-called TOPscore, which incorporates information about the TSS heterogeneity and the length of the TOP motif. In addition, genome-wide precise 5′ end mapping studies have suggested that, besides the canonical 5′TOP genes, the transcriptome contains more than a thousand additional mRNAs that encode 5′TOP motifs [20].

**Fig. 3:**
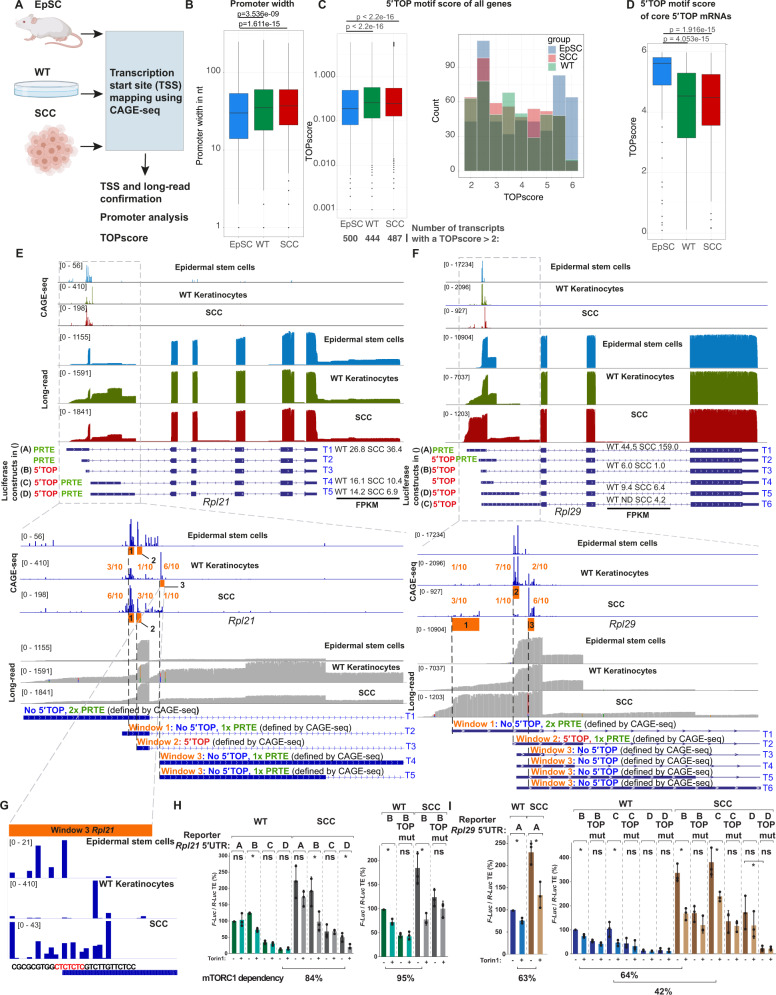
5′UTR isoform switches in *Rpl21* and *Rpl29* increase their translational efficiency in squamous cell carcinomas. **A** Outline of the cap analysis of gene expression (CAGE-seq) strategy to map transcription start sites (TSS) in SCA-1+ epidermal stem cells (EpSC), wild-type keratinocytes (WT) and cultured squamous cell carcinoma cells (SCC^c^). **B** Promoter width is genome-wide increased in WT and SCC^c^. Promoter width was computed using the CAGEr pipeline. *P*-values indicate a Wilcoxon test comparing the different promoter widths. **C** WT and SCC^c^ have a higher median TOP score but fewer transcripts with a TOP score >2. TOP scores were calculated using our CAGE-seq data and the previously reported TOP score script [10]. Left panel shows the overall distribution and the total number of transcripts with TOP scores above 2 (below). Right panel displays the distribution of the transcripts with TOP scores above 2. Only genes with an average of >500 reads in the CAGE-seq dataset were included. *P*-values indicate a Wilcoxon test comparing the TOP score distributions. **D** TOP scores for the 97 core 5′TOP mRNAs in SCA-1+ epidermal stem cells (EpSC), wild-type keratinocytes (WT) and cultured squamous cell carcinoma cells (SCC^c^). *P*-values indicate a Wilcoxon test comparing the different TOP score distributions. **E**, **F** Representation of the Nanopore long-read and CAGE-seq data set for the two ribosomal genes *Rpl21* and *Rpl29* and their transcript annotation. Red 5′TOP indicates a transcript containing a 5′ terminal oligopyrimidine motif, defined by a C at position +1 and an unbroken series of 4–16 pyrimidines. PRTE: pyrimidine-rich translational element, as defined by a stretch of 9 consecutive pyrimidines and an invariant uridine at position 6 in the 5′UTR. The letter next to the different transcripts refers to the luciferase constructs tested in **H** and **I**. Lower panels: orange windows indicate the major TSS regions in the EpSC, WT and SCC^c^. The fractions indicate the distribution of WT and SCC^c^ CAGE-seq reads in these three windows. The 5′TOP, no 5′TOP or PRTE labeling refers to the major CAGE peaks and not the annotated transcript, as further exemplified in **G**. FPKM: fragments per kilobase million, as quantified in the long-read sequencing data. **G** CAGE-seq read distribution within window 3 of *Rpl21*. Even though the annotated transcript starts with a 5′TOP motif, WT keratinocytes exhibit a major TSS that begins only 8 nucleotides downstream of the annotated transcript and does not include a 5′TOP motif. **H**, **I** The different *Rpl21* and *Rpl29* 5′UTR isoforms show a wide range of translational efficiencies. Wild-type (WT) or squamous cell carcinomas (SCC^c^) were transfected with an *Rpl21* or *Rpl29* 5′UTR::Firefly-luciferase and a control 5′UTR::*Renilla*-luciferase plasmid and treated for 3 h with 500 nM of the mTORC1 inhibitor Torin 1 (+) or DMSO (−) before harvesting. The labeling refers to the transcript isoform labeling in **E**, **F**, upper panels. First construct in WT was set to 100%. TOP mut indicates that the entire 5′TOP motif in the respective construct was mutated. Data represent the average of 3 independent experiments ±s.d. Asterisks indicate a *p*-value <0.05 using an ANOVA test. mTORC1 dependency was calculated as follows: (SCC no Torin 1 - SCC Torin 1)/(SCC no Torin 1 - WT Torin 1).

To confirm the precise TSS of the transcripts identified by our long-read sequencing data, we next captured capped 5′ ends of EpSCs, WT keratinocytes and SCC^c^ transcripts for cap analysis of gene expression (CAGE-seq) (Fig. 3A) [21]. The large-scale analysis of the 5′ transcript ends not only allows the determination of the exact TSS, but can also predict promoter regions and the exact TOP scores in the different cell types. Mammalian promoters can be subdivided into two main groups: TATA box-enriched sharp and well-defined promoters and more plastic, CpG-rich, broad promoters [22]. Using the CAGEr pipeline [23], we found that WT and SCC^c^ exhibited overall an increased promoter width compared to EpSCs, indicating the more frequent use of plastic TSSes in differentiated cells compared to stem cells (Fig. 3B).

To assess the usage of TOP motifs in our CAGE-seq dataset, we next employed the TOPscore metric, which quantifies the length of consecutive C/U and the peak height at each position of a 5′CAGE-seq end [10]. We found that WT and SCC^c^ both showed overall a higher median TOPscore (Fig. 3C) than EpSCs, suggesting that WT and SCC^c^ use genome-wide more frequently 5′motifs subject to mTORC1/LARP1-mediated translational regulation. The core 5′TOP mRNAs have previously been reported to span a TOP score between 2 and 6 [10]. Thus, we also assessed the number of mRNAs with more homogenous usage of 5′TOP motifs resulting in TOP scores >2. Despite the lower median TOP score of EpSCs, the number of transcripts with a TOP score >2 was higher in EpSCs (Fig. 3C, numbers below left panel). In many cases, the broader promoter region in WT and SCC^c^ (Fig. 3B) also resulted in a less strict usage of the potential 5′TOP motif, a feature that was also evident in the cohort of “core” 5′TOP mRNAs (Fig. 3D).

To experimentally test how individual 5′UTR isoforms impact the translational efficiency of an mRNA, we next focused on two ribosomal protein transcripts, *Rpl21* and *Rpl29*, which surfaced in our SplAdder analysis and showed distinct 5′UTR representations in the long-read Nanopore sequencing data as also highlighted by the sashimi plots (Fig. 3E–G, Supplementary Fig. 1F, G). *Rpl21* and *Rpl29* express several isoforms differing in their 5′UTR sequences but contain identical coding and 3′UTR sequences. In addition, the expression level of several *Rpl21* and *Rpl29* 5′UTR isoforms was significantly changed in SCC^c^ as quantified by the short-read RNA-seq data and the Ensembl annotation (Supplementary Fig. 1E). Both *Rpl21* and *Rpl29*, also showed increased overall translational efficiencies in SCC^c^ compared to wild-type keratinocytes (log_2_ fold change of 1.38 and 1.25). We revised the 5′UTR annotation according to our de novo assembled transcriptome and the CAGE-seq data. As opposed to other ribosomal proteins with sharp promoters such as, for instance, *Rpl10* (Supplementary Fig. 1H), *Rpl21* and *Rpl29* have broad promoter regions. We cloned the main transcript 5′UTR isoforms into Firefly luciferase constructs to express them in wild-type keratinocytes and SCC^c^ (Fig. 3H, I). To calculate translational efficiencies, we then measured *Rpl-*5′UTR Firefly and control-5′UTR *Renilla* luciferase activities and performed quantitative real-time PCR to assess mRNA levels. In addition, to address mTORC1 dependency of TOP motif-containing reporters experimentally, we treated the cells with the mTOR inhibitor Torin 1 or additionally mutated the 5′TOP motif.

In line with the translational efficiency quantification by ribosome profiling and RNA-seq, SCC^c^ generally showed higher TEs of the 5′UTR constructs (Fig. 3H, I). Since CAGE-seq datasets are more quantitative than long-read sequencing data, we assessed the CAGE-seq peak distribution for *Rpl21*, which indicated a relative switch from window 3 in WT keratinocytes toward windows 1 + 2 in SCC^c^ (Fig. 3E, F). This TSS switch was accompanied by a higher TE in SCC^c^ as we found that the corresponding luciferase construct B was more efficiently translated than constructs C and D, corresponding to the WT window 3 (Fig. 3H, left panel, constructs B vs C or D). However, the analysis also demonstrated that, at least for broad promoters, 5′UTR constructs cannot always accurately model their regulation because small differences in the exact TSS can define its nature and regulation as a 5′TOP motif. This was particularly evident for *Rpl21* window 3, which – instead of the annotated 5′TOP motif TSS – starts in WT 8 nucleotides further downstream with a non-TOP motif and switches to a broader peak distribution in SCC^c^, which now include 5′TOP motifs (Fig. 3G). Nevertheless, we could confirm the mTORC1-dependency of the 5′TOP motif-containing constructs *Rpl21-B/D* and *Rpl29-B/C/D* in SCC^c^ by Torin 1 treatment or mutation of the TOP sequence (Fig. 3H, I, *Rpl29-D* TE reduced by TOP mutation). The TOP-less construct *Rpl21-A* did not show any mTORC1-dependency, while *Rpl21-C* exhibited no reduction in translational efficiency by Torin 1 despite a 5′TOP motif (Fig. 3H, I). Moreover, a previous study indicated that >90% of mTORC1-targeted mRNAs contain either a 5′TOP or a so-called pyrimidine-rich translational element (PRTE), suggesting that either a 5′TOP or a PRTEs is a predictor of mTORC1-dependency [6]. Indeed, while the TOP-less construct *Rpl21-A* showed no mTORC1 sensitivity despite a PRTE, we found that the TOP-less *Rpl29-A* construct contained a PRTE motif, providing an explanation for its mTORC sensitivity in the absence of a 5’TOP.

**Fig. 4:**
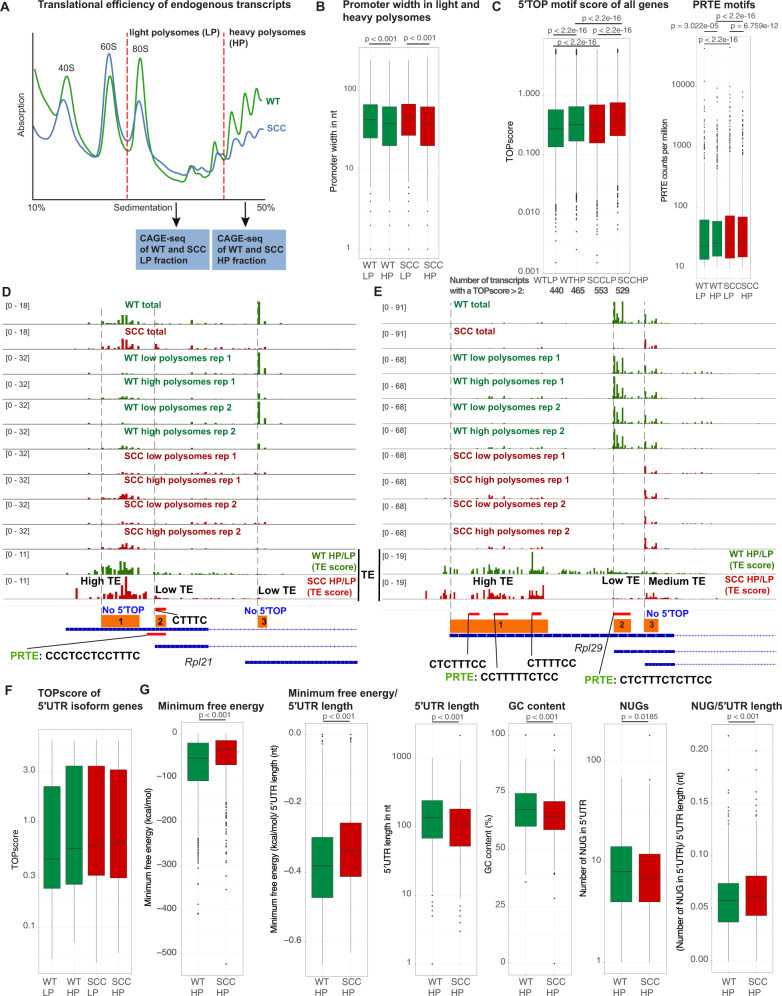
Squamous cell carcinomas express 5′UTR isoforms with high translational potential. **A** Outline of the combined polysome profiling and cap analysis of gene expression (CAGE-seq) strategy to map transcription start sites (TSS) in wild-type keratinocytes (WT) and cultured squamous cell carcinoma cells (SCC^c^). WT and SCC^c^ lysates were subjected to sucrose density gradient fractionations and light polysome (LP) and heavy polysome (HP) fractions were collected. RNA from the LP and HP fractions was isolated and CAGE libraries were prepared. Data represent the average of 2 independent experiments. **B** Promoter width is decreased in heavy polysome fractions in both WT and SCC^c^. **C** SCC^c^ transcripts have higher median TOP and PRTE scores in both the light and the heavy polysome fractions. In addition, SCC^c^ have a higher number of transcripts with a TOP score >2 (total transcript numbers below the graph). TOP scores were calculated using WT and SCC^c^ LP and HP CAGE-seq data and the previously reported TOP score script [10]. PRTE scores were determined by searching for PRTE motifs in WT and SCC^c^ LP and HP CAGE-seq data, normalized by the total number of reads. Median PRTE scores were 22.9 (WT LP), 25.8 (WT HP), 24.9 (SCC^c^ LP) and 26.9 (SCC^c^ HP). PRTE-containing 5′UTRs were defined by a PRTE score >10. PRTE: pyrimidine-rich translational element, defined by a stretch of 9 consecutive pyrimidines and an invariant uridine at position 6 in the 5′UTR. Data represent the average of 2 independent CAGE-seq experiments. *P*-values indicate a Wilcoxon test comparing the TOP score distributions or PRTE scores. **D**, **E** CAGE-seq peaks of total, light and heavy polysome fractions in the main three WT and SCC^c^ TSS windows. Data were normalized and group-autoscaled to directly compare parallel CAGE-seq experiments. The last two rows display the ratios of HP/LP in WT and SCC^c^ as a proxy for translational efficiency of the corresponding TSS. Regions were subdivided into low (ratio < 1), middle (ratio 1–3) and high TE (ratio > 3). Red bars indicate potential 5′TOP TSS with the corresponding sequence or a PRTE. PRTE: pyrimidine-rich translational element, defined by a stretch of 9 consecutive pyrimidines and an invariant uridine at position 6 in the 5′UTR. **F** TOP scores of light and heavy polysome fractions for the cohort of 5′UTR isoforms. **G** Heavy polysome 5′UTRs in SCC^c^ are less structured and show fewer potential upstream open reading frames. WT and SCC^c^ HP CAGE transcription start sites (ctss) were grouped into tag cluster promoter regions and directly compared using DESeq2. For the significantly up- and downregulated SCC^c^ clusters, corresponding 5′UTRs isoforms were subsequently extracted. Upregulated 5′UTRs in SCC^c^ and WT were compared for the minimum free energy, length, GC content and potential 5′UTR uORF initiation sites (NUGs). *P*-values indicate a Wilcoxon test.

Because of the above-mentioned caveat of using luciferase constructs to understand broad promoters and the potential bias from overexpression upon transient transfection, we next sought to assess endogenous transcripts. To this end, we subjected WT and SCC^c^ to sucrose density gradient fractionation and collected light (up to 3 ribosomes) and heavy polysome (≥4 ribosomes) fractions to represent low and high translational efficiencies. We then similarly performed CAGE-seq to annotate the precise TSSes of mRNAs isolated from light and heavy polysome fractions (Fig. 4A). In line with a previous report using *O*-propargyl- puromycin (OPP) incorporation assays, SCC^c^ exhibited lower polysome fractions compared to WT keratinocytes indicating lower overall protein synthesis rates, likely due to increased phosphorylation of eIF2α [19]. Despite lower overall protein synthesis rates, we confirmed that SCC^c^ have higher mTORC1 activity levels by assessing the phosphorylation of two classic downstream targets, 4E-BP1 and S6K (Supplementary Fig. 2A). Thus, mTORC1-driven effects can increase the translational efficiency of specific cohorts of genes even in the face of global translation reduction. Generally, highly translated transcripts showed shorter promoter width than transcripts in the light polysome fraction (Fig. 4B). We next analyzed genome-wide 5′TOP and PRTE motif usage in light and heavy polysome fractions based on our exact CAGE-seq peak distribution in the promoter regions. We observed that SCC^c^ had higher TOP and PRTE scores than WT in both light and heavy polysome fractions (Fig. 4C, Supplementary Fig. 2E). This was also evident in the number of transcripts with a TOPscore >2 (Fig. 4C) [10]. In addition, as expected, heavy polysome fractions showed higher TOP and PRTE scores than light polysome fractions in both WT and SCC^c^. These data suggest that SCC^c^ switch genome-wide towards higher 5′TOP and PRTE motif usage and regulation by mTORC1 and that higher TOP and PRTE scores generally correlate with higher translational efficiency in both WT and SCC^c^.

We next examined the 5′UTR isoform genes *Rpl21* and *Rpl29*. While the two main WT TSS windows, *Rpl21-3* and *Rpl29-2*, exhibited low TE based on the HP/LP ratios, SCC^c^ switched to increased relative usage of the TSS windows *Rpl21-1, Rpl29-1* and *Rpl29-3* with high and medium TEs (Fig. 4D, E). Despite the high TE in SCC^c^*, Rpl21* TSS window 1 contains no 5′TOP motifs. The annotated transcript corresponding to the other high TE *Rpl29-1* window does also not start with a 5′TOP motif. However, its broad TSS window contains 3 stretches of 5′TOP motifs, which all show high TEs (Fig. 4D, E). This illustrates the importance of not solely relying on annotated TSS because, within broad promoter regions, additional 5′TOP motifs could be utilized. In addition, since the presence of either a 5′TOP or a PRTE is a strong predictor for mTORC1 sensitivity [6], we also searched for PRTE motifs. We found that the *Rpl21-1* and *Rpl29-1* windows with high TE both contained PRTE motifs, suggesting that PRTE motifs could indeed serve as a predictor for a transcript’s mTORC1-dependency. Furthermore, the TOP scores of all 73 5′UTR isoforms in light and heavy polysome fractions showed a trend towards higher TOP scores in SCC^c^ and in the heavy polysomes, but were too heterogenous to show statistically significant TOP score alterations (Fig. 4F). To summarize, as exemplified by *Rpl21* and *Rpl29*, these transcripts suggest that SCC^c^ generally switch to TSSes with higher TE, which genome-wide exploit more frequently 5′TOP and PRTE motifs based on the CAGE-seq data (Fig. 4C).

To systematically analyze the features of highly translated 5′UTR isoforms besides 5′TOP and PRTE motifs, we next directly compared the WT and SCC^c^ high polysome fractions. We clustered CAGE transcription start sites (ctss) into tag cluster promoter regions within the CAGEr pipeline and identified significantly altered clusters between SCC^c^ and WT by DESeq2. We then extracted the 5′UTR isoforms corresponding to these cluster promoter regions and examined different features of these isoforms. Highly translated SCC^c^ 5′UTRs showed less negative folding free energy, even when normalizing to length and exhibited lower GC content (Fig. 4G), features that are generally implicated in decreased 5′UTR RNA secondary structures. Although the potential 5′ uORF translation start sites as defined by NUG density (AUG, CUG, GUG or UUG) was higher in SCC^c^, because of the 5′UTR length difference, we found a decreased total number of potential upstream open reading frames (uORFs) in highly translated SCC^c^ transcripts, (Fig. 4G). These results demonstrate that, in addition to higher 5′TOP and PRTE motifs usage, SCC^c^ generally also express 5′UTR isoforms that exhibit features implicated in more efficient scanning and higher translation initiation rates.

**Fig. 5:**
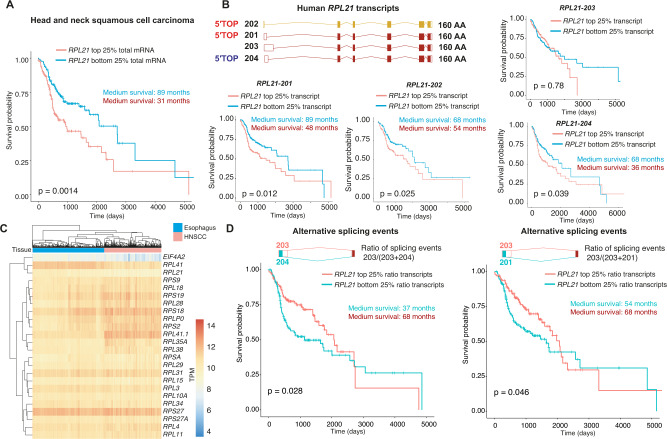
The 5′TOP motif-containing, but not the TOP-less, *RPL21* isoforms correlate with overall survival in head and neck squamous cell carcinoma (HNSCC) patients. **A** Increased *RPL21* mRNA levels correlate with shorter overall survival of human head and neck squamous cell carcinoma (HNSCC) patients. *RPL21* top and bottom quartile mRNA expression in TCGA HNSCC patients′ samples (*n* = 519). Cox regression hazard ratio 1.4629. **B** Increased *RPL21* TOP motif-containing transcripts 201 and 202, but not the TOP-less transcript 203, correlate with shorter overall survival of head and neck squamous cell carcinoma (HNSCC) patients. *RPL21* top and bottom quartile transcript expression in TCGA HNSCC patients′ samples (*n* = 519). The 4 main human *RPL21* isoforms are depicted in the upper panel, all isoforms code for the identical 160 amino acid RPL21 protein. Two transcripts contain a classic 5′TOP motif (red), one contains a TOP-like motif (blue) and one transcript does not express a 5′TOP motif. **C** mRNA expression levels of the differentially spliced translation-related genes in TCGA head and neck squamous cell carcinoma (HNSCC) patients′ samples (*n* = 519) and non-diseased esophageal tissue (non-diseased pharynx samples were not available). **D** Alternative splicing events significantly stratify head and neck squamous cell carcinoma patient survival. The alternative splicing events between the two transcripts correlating with survival, *RPL21-201* and *RPL21-204*, and the transcript that did not correlate with survival *(RPL21-203)* were directly compared in head and neck squamous cell carcinoma patients′ samples (*n* = 519). The ratio between the alternative splicing events was calculated and the overall survival of the top and bottom quartile alternative splicing events was assessed.

Finally, to determine whether 5′UTR isoform switches could also play a role in human cancer, we assessed *RPL21* and *RPL29* levels in 519 human head and neck squamous cell carcinoma (HNSCC) patients. Increased *RPL21*, but not *RPL29*, total mRNA levels correlated with shorter overall survival in HNSCC patients (Fig. 5A–C, Supplementary Figs. 2B, 3A, B). The human *RPL21* gene contains 4 main isoforms that encode identical proteins and only differ by their 5′UTR. Intriguingly, we found that the two TOP motif-containing transcripts correlated with overall survival in human HNSCC patients (Fig. 5B, Supplementary Fig. 4). For instance, HNSCC patients with low expression of the *RPL21-201* isoform showed an almost 3.5 years longer medium survival than HNSCC patients with high expression of this 5′UTR isoform (89 vs. 48 months). In contrast, the transcript without a TOP motif showed no correlation with survival, despite coding for the same protein, while the transcript with a TOP-like motif weakly correlated with survival in HNSCC patients (Fig. 5B, Supplementary Fig. 4). This was also true when we directly assessed specific alternative splicing events in HNSCC patients. The direct comparison and ratio of alternative 5′UTR splicing events between the transcripts *RPL21-201/204* correlating with survival and the non-correlating transcript *RPL21-203* also significantly stratified the overall survival of HNSCC patients (Fig. 5D, Supplementary Fig. 4). Collectively, these observations suggest that 5′UTR isoform switches may also be relevant for disease progression in cancer patients and strongly warrant further isoform-specific analyses in human cancer datasets.

## Discussion

Transcription and translation together determine ~90% of cellular protein abundance [24] and it is a compelling question to what extent these two processes can be directly coupled in different biological contexts. In this short report, we carried out Nanopore long-read sequencing and polysome-associated CAGE-sequencing to document the UTR isoform landscape and the complexity of promoter regions of in vivo epidermal stem cells and cultured squamous cell carcinoma cells (SCC^c^). We provide the combined dataset and the de novo assembled, curated transcriptomes on an easily accessible genome browser as a resource. We show that a small cohort of 5′UTR isoforms, differentially expressed in SCC^c^, can alter the translational efficiency of the corresponding coding sequence. Our analyses revealed that highly translated SCC^c^ transcripts exhibit genome-wide increased use of 5′UTR motifs that are known to drive mTORC1-dependent translation of the mRNA. Notably, increased expression of TOP motif-containing, but not the TOP-less, *RPL21* transcripts strongly correlated with shorter overall survival in head and neck squamous cell carcinomas (HNSCC), indicating that the precise configuration of the 5′UTR isoforms may be relevant for disease progression.

Our observations suggest that for a cohort of translation-related genes, rather the type of 5′UTR isoform instead of absolute mRNA levels would determine cellular protein levels. In keeping with this notion, while the genome-wide median correlation between mRNA and protein levels in human HNSCC patients was r_s_ = 0.52, ribosomal protein transcripts, including *Rpl21* and *Rpl29*, exhibited particularly poor correlations (Supplementary Fig. 2C, D) [25]. This observation indicates that post-transcriptional regulatory mechanisms, including the translational potential of 5′UTR isoforms, are critical in determining cellular ribosomal protein levels in human cancer. Nevertheless, given the relatively small cohort of 5′UTR isoform switches, our observations also suggest that the network of translational regulators is genome-wide still the main factor determining protein synthesis rates.

5′UTR isoform switches have emerged as an elegant non-canonical mode of gene expression regulation [1–3, 26]. Our findings add yet another simple mechanism to the 5′UTR landscape and provide a paradigm in which the exposing or masking TOP and PRTE motifs from the 5′UTR can markedly alter the translational efficiency of the mRNA coding sequence in cancer. Translational efficiency changes could be achieved by relatively small changes in the exact TSS that can expose additional 5′UTR features or modify the presence of 5′TOP and PRTE motifs to set the dependency on mTORC1-dependent nutrient sensing [6, 7]. Given that alternative splicing and TSSes are not only widespread [11, 12], but also given the heterogeneity of TSSes for broad promoter genes, switching between 5′UTR isoforms with different sets of post-transcriptional regulatory elements such as uORFs, RBP binding motifs, 5′TOP or PRTE motifs could be used during different stages of tumorigenesis to directly encode the mRNA′s translational potential in its 5′UTR.

## Material & methods

### Genome browser

Nanopore long-read RNA sequencing, CAGE data and de novo transcriptomes are available in the UCSC genome browser under the following link: https://genome-euro.ucsc.edu/s/umeshghosh/cage_ont. Data on the genome browser is shown as TPM (transcripts per kilobase million) for Nanopore and tags per million in case of CAGE. Note that the genome browser uses bigwig and not bam files, which can result in a slightly different representation of the data. Updated links for the genome browser will be provided on the github page (https://github.com/ugdastider/long_read_paper).

### Nanopore long-read RNA sequencing

RNA was isolated using TRIzol LS (Thermo Fisher, 10296010) and the Direct-zol™ RNA Miniprep Kit (Zymo Research, R2050). The samples were then used for library preparation following the manufacturer′s protocol of SQK-PCS109 (Oxford NANOPORE Technologies, Oxford Science Park, UK). Briefly, total RNA was polyA enriched using oligo dT, annealed with primers, and reverse transcribed. Following template switching, PCR with rapid attachment primers was performed and rapid 1D sequencing adapters were attached. The library was then sequenced on PromethION (Oxford NANOPORE Technologies, UK).

### Isolation of adult mid-telogen EpSCs

Female C57BL6 mice were obtained from Janvier. EpSCs were isolated from telogen back skin collected from P56-60 mice, as previously described [27] with the following changes: fat and muscle tissue were removed from back skin using a scalpel. The skin was incubated in 0.5% Trypsin-EDTA (10X; Gibco,15400054) for 25 min at 37 °C on an orbital shaker. A single-cell suspension was then obtained by scraping the skin with a scalpel followed by neutralizing the trypsin by adding 1X PBS buffer containing 2% chelexed FBS (PBS-FBS(-)) (Gibco; 10010-015). The resulting cell suspension was then filtered through 70 µm and 40 μm cell strainers (Corning; 431750, 431751) and spun down. EpSCs were isolated using magnetic-associated cell sorting using Anti-SCA-1 microbeads (Miltenyi Biotec, 130-106-641) and a MACS MultiStand system (Miltenyi Biotec) together with MS columns (Miltenyi Biotec, 130-042-201). The resulting SCA-1+ EpSCs were spun down and resuspended in Trizol LS (Thermo Fisher, 102960-10). RNA was isolated using the Direct-zol™ RNA MiniPrep kit (Zymo Research, R2050) and concentration was determined using the Qubit^TM^ RNA BR assay kit (Invitrogen, Q10210). All animal procedures were approved by the Veterinary Office of the Canton of Zurich, Switzerland (License ZH233/2019).

### In vitro cell culture experiments

Cell lines were cultured in 0.05 mM Ca^2+^ E-media, made in house from DMEM/F12 medium supplemented with 15% chelated FBS, 5 μg/mL insulin, 5 μg/mL transferrin, 2 nM triiodothyroxine, 40 μg/mL hydrocortisone and 10 nM cholera toxin. Cells were grown under standard tissue culture conditions, 37 °C and 5% CO_2_. *Hras*^*G12V*^*; Tgfbr2* knockout cell line was previously generated [15]. Newborn, primary mouse epidermal keratinocytes from wild-type mice were cultured on 3T3-S2 feeder layer in 0.05 mM Ca^2+^ E-media supplemented with 15% serum [27]. Cell lines were tested for mycoplasma infection every 3 months using the Mycoplasma PCR Detection kit (Sigma, D9307).

### Luciferase assays

0.25 × 10^6^ cells were plated in a total volume of 2 ml 0.05 mM Ca^2+^ E-media in 6-well plates 24 h before transfection. The transfection mixtures contained 1.5 µg Rpl29/Rpl21-F-Luc and 0.5 µg control R-Luc plasmid DNA. All transfections were performed using Lipofectamine 2000. 2 h after transfection, cells were treated with 500 nM Torin 1 or DMSO and harvested 5 h after transfection. Cells were briefly washed with PBS and lysed in passive lysis buffer. Firefly and *Renilla* luciferase activities were measured at room temperature using the Dual-Luciferase reporter assay system (Promega, E1980) and a Tecan Infinite M1000Pro instrument. All luciferase experiments were performed in biological triplicates.

### Quantitative real-time PCR

For RNA extraction, cells were resuspended in TRIzol LS (Thermo Fisher, 10296010) and extracted using chloroform. The aqueous phase was then precipitated in isopropanol, pelleted, and resuspended in H_2_O. For cDNA synthesis 0.5 µg total RNA was mixed with 0.5 µg of random hexamer primers (N_6_) and denatured at 72 °C for 5 min. Subsequently, a reaction mixture containing 1x SSIII RT buffer, 1 mM dNTPs, 5 mM DTT, and 0.5 µl SSIII (Invitrogen, 18080093) was added to reach a total volume of 20 µl. The RT reaction was performed at 55 °C for 1 h and inactivated for 10 min at 70 °C. The qPCR was performed in a final concentration of 1x iTaq Universal SYBR Green Supermix (Biorad, 1725121), 0.4 µM primer each, and 1 µl of the cDNA in a total volume of 10 µl.

### Bioinformatic analyses

Sample processing and analysis were performed on an Ubuntu 18.04.5 cluster with 32 cores and 128GB RAM. Standard parameters were used for all software unless stated otherwise, Python version is 3.8.6 unless indicated otherwise.

### Processing of Nanopore long-read data

The long-read sequencing data were processed by Nextflow nanoseq v1.1 [28] and a custom pipeline. The quality of the raw fastq files and the sequencing was assessed by the FastQC and NanoPlot [29] programs. Alignment to the mouse reference genome Gencode GRCm38 version M25 was carried out by minimap2, which can do both spliced and unspliced alignments. Custom transcriptome reference assembly and expression quantification were performed by StringTie2 [16]. Furthermore, the transcriptomes were merged and compared by GFFcompare v.0.11.2 [30]. Nanopore long-read data are available on the GEO GSE179525. Wild-type keratinocytes: GSM5419823. Squamous cell carcinomas: GSM5419824. Epidermal stem cells: GSM5419822.

### Processing of short-read RNA-seq data

We utilized the Nextflow RNA-seq pipeline [28] and custom scripts to process the short-read RNA-seq data. The quality of the fastq files was assessed by the FastQC program. TrimGalore was used to perform quality and adapter trimming of the sequencing data. The reads were aligned to the GRCm38 reference by STAR v2.6.1 [31]. Transcriptome quantification was carried out by Salmon [32]. BigWig files were created by BEDTools for visualization of coverage tracks. Furthermore, MultiQC was used to carry out quality control of all the analysis pipelines.

### Differential expression

The raw gene and transcript level counts obtained were processed by DESeq2 to call the differentially expressed (DE) genes between various conditions [33]. Hierarchical clustering and PCA plots after variance stabilizing transformation (vst) normalization of the top 500 most variable genes were used to detect any outlier samples. First, the count data were normalized by the median of ratios method. Next, the dispersion or biological variance was estimated. A generalized linear model was fitted for each gene to detect differentially expressed (DE) genes. The p-values obtained by the Wald test were corrected by the Benjamini–Hochberg multiple testing procedure. DE genes with FDR cut-off <0.05 were used for further analysis.

### Processing of ribosome profiling samples

Ribosome profiling samples were processed as described in the short-read RNA-seq section above, with the addition of a filtering step after the adapter trimming. Only reads not aligning to a merged transcriptome consisting of ribosomal, mitochondrial, or tRNA [34] sequences using Bowtie2 v2.4.1 [35] were processed further.

### Ribosome profiling and short-read RNA sequencing samples

Ribosome profiling and short-read RNA sequencing data were used from a previously published study [19] with matched wild-type keratinocyte and *Hras*^*(G12V);*^ *Tgfbr2* null squamous cell carcinoma samples, which are available on the GEO GSE83332 and GSE179525. We used the following samples: Wild-type keratinocytes: S11, S13 and S14 (Ribosome profiling, GSM2199591, GSM2199593, GSM2199594) and S27 and S28 (short-read RNA-seq, GSM2199607, GSM2199608).

Squamous cell carcinomas: samples S23, sample 197 and S24 (Ribosome profiling, GSM2199603, GSM5419825, GSM2199604) and samples 211 and 212 (short-read RNA-seq, GSM5419826, GSM5419827).

Epidermal stem cells: samples S7 and S8 (short-read RNA-seq, GSM2199587, GSM2199588), which are P4 epidermis samples enriched for epidermal stem cells. Note that ribosome profiling data were not available for P60 SCA-1+ epidermal stem cells.

### Curated transcriptome

For the curated transcriptome, long-read isoform quantification and characterization were also performed by SQANTI3 [36], using short-read RNA-seq samples in a sequential manner. First, the whole StringTie assembled transcriptome was used to quantify isoforms and subsequently subjected to the SQANTI3 filtering to remove not well-supported junctions and antisense transcripts passively produced by the ONT1D procedure. The obtained filtered annotation was used to create a new filtered transcriptome and re-map samples to increase mapping rates and counts. Functional annotations were transferred from a pre-computed tappAS annotation file [37] based on PacBio sequencing by facilitating the IsoAnnotLite (v2.6) option of SQANTI3.

### Translational efficiency

Genomic reads of RNA-seq and ribosome profiling samples were quantified by Plastid v0.4.7 [38] (Python 2.7) over extracted exon and coding sequences (CDS) regions, respectively, as outlined previously [19]. Translational efficiency (TE) was computed in R v4.0.2 using the LRT-test of the DESeq2 package and full/reduced models as suggested by the plastid authors [38] for genes that showed rpkm (read per kilobase per million mapped reads) >1 in RNA-seq samples. For comparison with the SplAdder gene lists (Fig. 2C), we included TE calculations with a base mean >25. Note that raw counts were used for DESeq2 and not rpkm as in previous TE calculations. Therefore differences in rpkm between samples are normalized out.

### Differential isoform events

Alternative splicing events were computed by SplAdder v2.4.3 [17] using maximum level 3 confidence parameters and the StringTie annotations and genome-mapped short-read RNA-seq samples. Results were subjected to a threshold filtering, requiring an adjusted *p*-value <0.05.

### Pathway enrichment

Selected genes were analyzed for gene ontology (GO) pathway enrichment using GSEA pre-ranked method which enables the analysis of up- and down-regulated genes simultaneously. This approach significantly improves the sensitivity of the gene set enrichment analysis. The overrepresentation analysis for the pathway enrichment was carried out by EnrichR and custom scripts.

### TCGA and GTEx data analysis

TCGA and GTEx data were obtained for HNSCC and esophagus from UCSC Xena Toil (xena.ucsc.edu). Survival analysis for TCGA HNSCC was performed by the survival and survminer library in R. A Cox Proportional Hazards regression model was used to fit the gene expression to survival to obtain the Hazard Ratio. Kaplan–Meier analysis was performed on groups with lower and upper quartiles of gene expression and the *p*-values were computed by a log-rank test.

### Sucrose gradient fractionation

To separate light and heavy polysome fractions of WT keratinocytes and SCC cells lysates were prepared in lysis buffer (20 mM Tris-HCl pH = 7.4, 150 mM NaCl, 5 mM MgCl_2_, 1% Triton X-100, 0.5% NP40, 1 mM DTT, 100 µg/ml cycloheximide) and polysomes separated on a 10–50% sucrose gradient in gradient buffer (20 mM Tris-HCl pH 7.4, 150 mM NaCl, 5 mM MgCl_2_, 100 µg/ml cycloheximide) for 2 h at 41000 rpm. Polysome fractions were collected using the Biocomp Density Gradient Fractionation System. To isolate RNA from sucrose fractions, the fractions of interest were pooled and mixed with 3 volumes Trizol LS (Ambion) and incubated for 10 min at room temperature to disrupt all protein-RNA interactions. Subsequently, samples were mixed with 4 volumes of 100% ethanol and purified using the Direct-zol™ RNA MiniPrep kit (Zymo Research, R2050).

### CAGE-seq

Qualities of total RNA were assessed by Bioanalyzer (Agilent) to ensure that RIN (RNA integrity number) is over 7.0. The cDNAs were synthesized from total RNA using random primers. The ribose diols in the 5′ cap structures of RNAs were oxidized, and then biotinylated. The biotinylated RNA/cDNAs were selected by streptavidin beads (cap-trapping). After RNA digestion by RNaseONE/H and adaptor ligation to both ends of cDNA, double-stranded cDNA libraries (CAGE libraries) were constructed. CAGE libraries were sequenced using single-end reads of 75nt on a NextSeq 500 instrument (Illumina). Obtained reads (CAGE tags) were mapped to the mm10 mouse genome. CAGE library preparation, and sequencing were performed by DNAFORM (Yokohama, Kanagawa, Japan). All CAGE-seq experiments from sucrose gradients to compare light and heavy polysome fractions were performed in biological duplicates. CAGE-seq experiments from total lysates were performed as one biological replicate. CAGE-seq data are available on the GEO GSE201308.

### Western blotting

Cells were washed with 1xPBS and lysed in protein sample buffer (100 mM Tris-HCl pH = 6.8, 4% SDS, 20% glycerol, 0.2 M DTT), boiled 5 min at 95 °C and vortexed to shear genomic DNA. Proteins were separated using SDS-PAGE and subsequently transferred onto a nitrocellulose membrane (GE Healthcare) by tank transfer. Membranes were blocked using 5% BSA, primary antibodies incubated overnight at 4 °C and secondary antibodies for 3 h at 4 °C. All western blots were developed with freshly mixed ECL solutions (GE Healthcare). Antibodies used in this study are the following: Phospho-p70 S6 Kinase (Thr389) from Cell Signaling (#9234), 4E-BP1 from Cell Signaling (#9452), Phospho-4E-BP1 (Thr37/46) from Cell Signaling (#2855), Tubulin from Sigma Aldrich (T6199). Western blot bands were quantified using ImageJ.

### Survival analysis of transcript usage

The alternative transcript usage in the TCGA HNSCC dataset was calculated using SUPPA2 [39]. First the relative transcript abundance of each transcript was calculated by psiPerIsoform command. Next PSI values for transcripts were combined with the survival data to identify the association of survival to top and bottom quartile patients based on the log-rank test.

### Analysis of CAGE data

CAGE data was processed by Nextflow cageseq pipeline [40], (https://nf-co.re/cageseq). First the 5′G was trimmed and reads were aligned to the mouse gencode M25 genome by STAR. Further processing of the bam files was carried out by the CAGEr package [23], (https://bioconductor.org/packages/release/bioc/html/CAGEr.html). The CTSS tag counts were calculated and normalized to tpm (transcripts per million) units. CTSS regions were clustered by distance based on “distclu” method. Promoter quantile width from 0.1 to 0.9 based on the tag clusters was calculated by the quantilePositions function. Differential expression analysis of tag counts from CAGEr was performed by DESeq2 [33]. Analysis of the features of the 5′UTR sequence (such as GC content, NUG content) was carried out by custom python scripts. The Vienna RNA python library was used for the minimum free energy (MFE) calculation [41]. We define sharp promoters as an interquartile range of <10 bp and broad promoters by an interquartile range of ≥10 bp.

### SQANTI quality control (QC) reports

SQANTI3 QC reports are available under the following link: https://github.com/ugdastider/long_read_paper/tree/main/sqanti

### TOPscore calculation

We obtained the TOPscores from the CAGE bam files using the tss_analyzer script from https://github.com/carsonthoreen/tss_tools [10]. The data were filtered to only include mRNAs with an average >500 CAGE-seq reads in the different samples.

### PRTE quantification

We searched the CAGE-seq reads mapped to the mouse genome to identify the presence of PRTE motifs. PRTE motifs were defined by a stretch of 9 nucleotides of C/T with a fixed T at position 6. For each gene, we counted the total number of reads mapping to the gene containing the PRTE motif by a custom Python script. The total PRTE counts were normalized to library size to counts per million mapped reads for each sample. We considered genes with >10 PRTE counts per million for further analysis.

## Supplementary information

Supplementary Figures

Supplementary Figure Legends

## Data Availability

All sequencing data generated in this study have been submitted to the NCBI Gene Expression Omnibus (GEO, https://www.ncbi.nlm.nih.gov/geo/) under accession numbers GSE201308 and GSE179525. Processed Nanopore long-read RNA sequencing, CAGE data and de novo transcriptomes are available in the UCSC genome browser under the following link: https://genome-euro.ucsc.edu/s/umeshghosh/cage_ont.
