# Supplementary material for: Monitoring the 5′UTR landscape reveals isoform switches to drive translational efficiencies in cancer
Source: Oncogene. 2022 Dec 23;42(9):638–50. doi: 10.1038/s41388-022-02578-2 (PMC9957725; doi:10.1038/s41388-022-02578-2)

Supplementary Figure 1

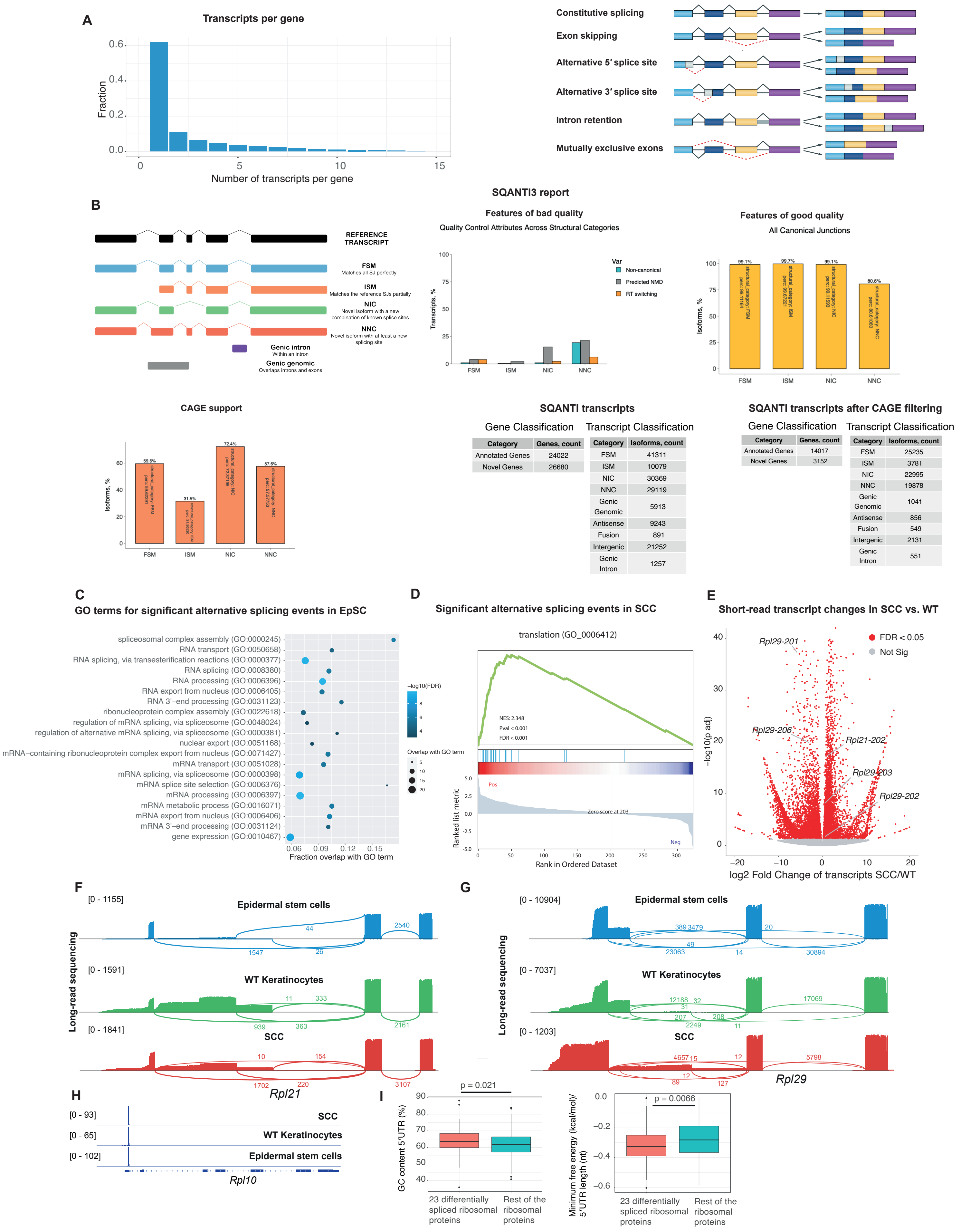

SQANTI transcripts — Gene Classification

Category	Genes, count
Annotated Genes	24022
Novel Genes	26680

SQANTI transcripts — Transcript Classification

Category	Isoforms, count
FSM	41311
ISM	10079
NIC	30369
NNC	29119
Genic Genomic	5913
Antisense	9243
Fusion	891
Intergenic	21252
Genic Intron	1257

SQANTI transcripts after CAGE filtering — Gene Classification

Category	Genes, count
Annotated Genes	14017
Novel Genes	3152

SQANTI transcripts after CAGE filtering — Transcript Classification

Category	Isoforms, count
FSM	25235
ISM	3781
NIC	22995
NNC	19878
Genic Genomic	1041
Antisense	856
Fusion	549
Intergenic	2131
Genic Intron	551

Supplementary Figure 2

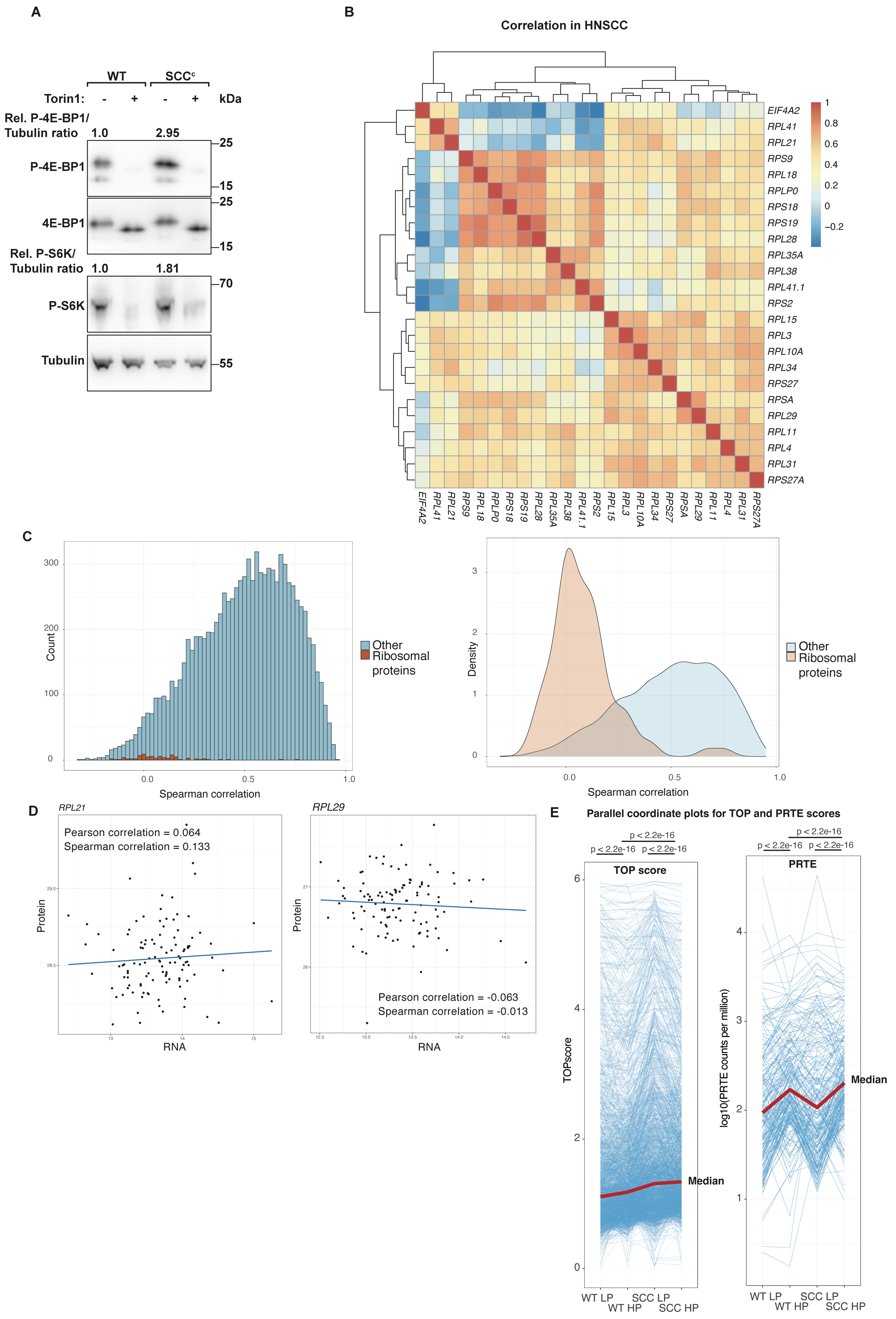

Supplementary Figure 3

A ***RPL21*** **levels and survival in human cancer**

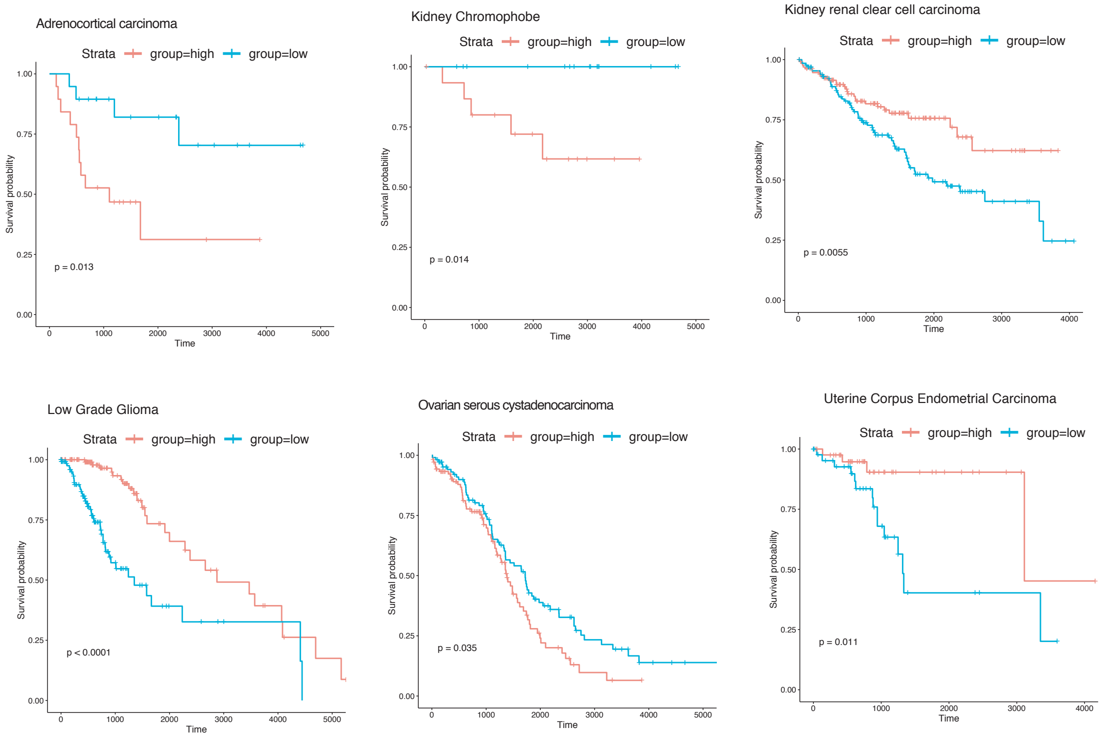

B ***RPL29*** **levels and survival in human cancer**

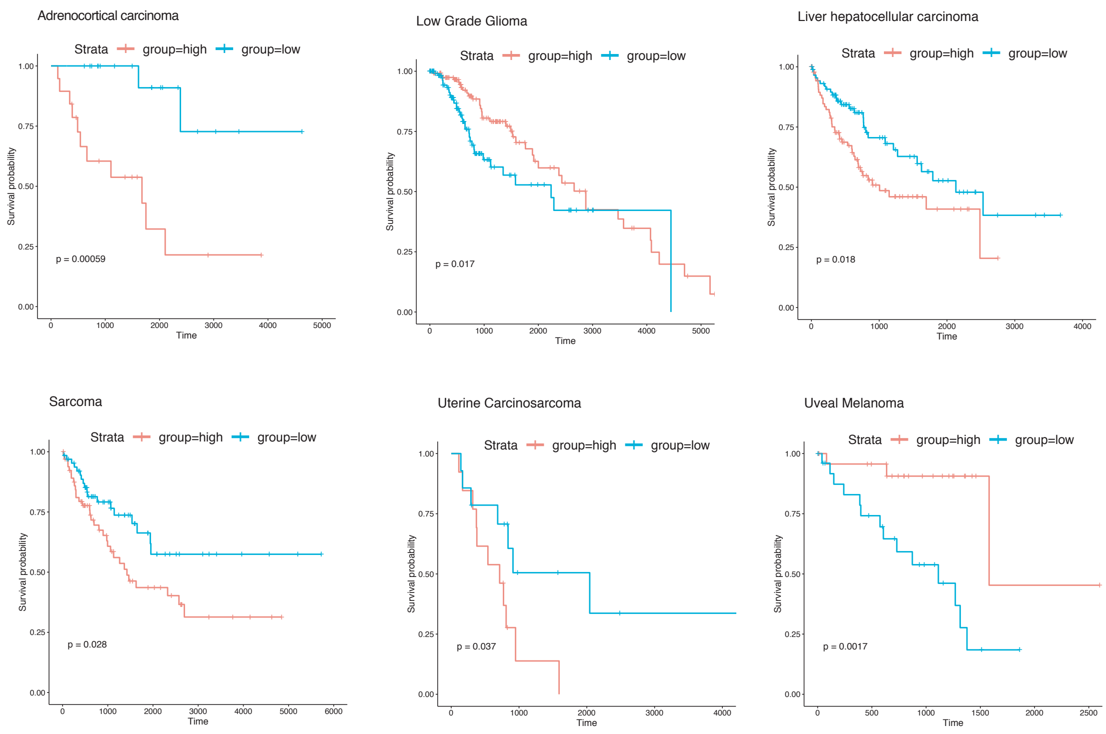

Supplementary Figure 4

A

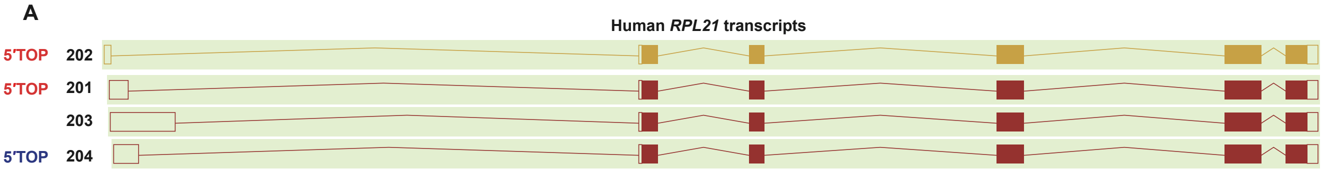

204 5′-GCGTGGCTCCCGGGCGCTAGGTGTTCGACAGACTT

B

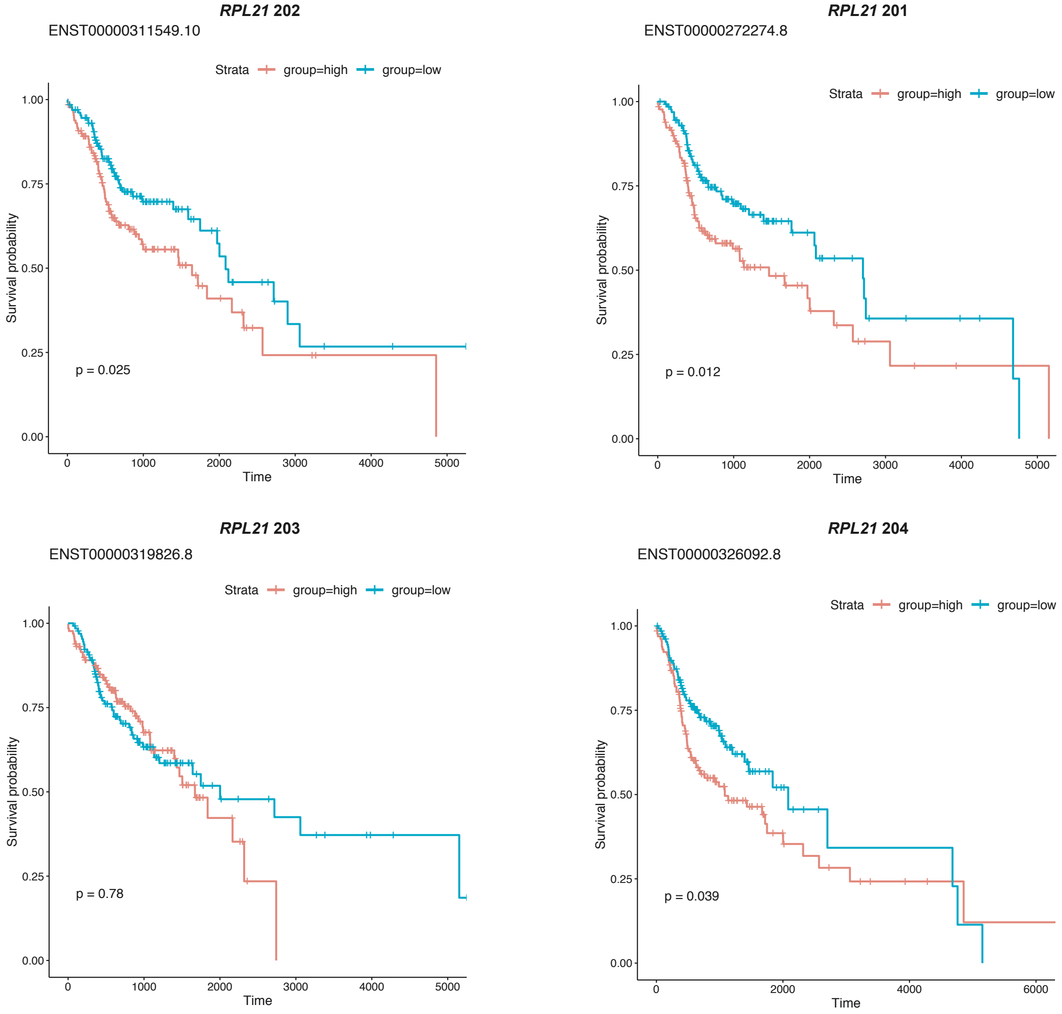

Supplement: Supplementary file 1 — Supplementary Figures [file 41388_2022_2578_MOESM1_ESM.pdf]
