# Supplementary material for: Monitoring the 5′UTR landscape reveals isoform switches to drive translational efficiencies in cancer
Source: Oncogene. 2022 Dec 23;42(9):638–50. doi: 10.1038/s41388-022-02578-2 (PMC9957725; doi:10.1038/s41388-022-02578-2)
Supplement: Supplementary file 2 — Supplementary Figure Legends [file 41388_2022_2578_MOESM2_ESM.pdf]

Supplementary Figure legends

Supplementary Fig. 1.

A. Left panel, number of transcripts per gene as identified by the StringTie pipeline. The right panel shows a diagram of the definition of the alternative splicing events of SplAdder.

B. SQANTI3 report showing features of good and bad quality in our long-read sequencing dataset and the percentage of transcripts supported by the CAGE-seq dataset. FSM: perfect match in splice junctions, ISM: matches splice junctions partially, NIC: novel isoform with a new combination of known splice junctions, NNC: novel isoform with at least a new splice site, SJ: splice junction, NMD: nonsense-mediated mRNA decay, RT switching: reverse transcriptase template switching.

C. Gene expression is the top gene ontology (GO) term for alternatively spliced genes also in epidermal stem cells. GO term analysis shows the top 20 GO term hits (alphabetically ordered) with their false discovery rate (FDR, blue tone) and overlap with the GO term gene list in numbers (size of circle) or the fraction (x-axis).

D. Enrichment score in Gene Set Enrichment Analysis (GSEA) analysis for the GO term translation for the genes differentially spliced in SCC^c (using the pre-ranked approach with log-fold splicing event changes in SCC over WT). P values were obtained by 1000 permutation tests to build a null distribution of enrichment scores. NES: normalized enrichment score.

E. Volcano plot for DESeq2 differential transcript expression analysis in squamous cell carcinomas (SCC^c) compared to wild-type keratinocytes (WT) reveals several *Rpl21/Rpl29* transcripts (Ensembl annotation) with significantly altered expression levels. The number next to the gene name refers to the Ensembl transcript ID.

F-G. Sashimi plots of *Rpl21* and *Rpl29* highlight the differential isoform usage of the transcript 5′UTRs in the Nanopore long-read sequencing data.

H. CAGE-seq peak distribution in *Rpl10* reveals a sharp homogenous promoter.

I. The 5′UTRs of the 23 ribosomal proteins with significantly altered splicing events in SCC^c show higher GC content and are less structured, as assessed by the minimum free energy divided by the 5′UTR length. P-values indicate a Wilcoxon test comparing the 23 ribosomal proteins vs. the rest of the ribosomal proteins.

Supplementary Fig. 2.

A. SCC^c show higher mTORC1 activity levels. Western blot shows two classic mTORC1 targets, phosphorylation of 4E-BP1 and S6K. Cells were treated for 3 h with 500 nM of the mTORC1 inhibitor Torin 1 (+) or DMSO (-) before harvesting. Relative P-4E-BP1/Tubulin and P-S6K/Tubulin levels were quantified using ImageJ.

B. Correlation between the mRNA levels in human TCGA head and neck squamous cell carcinoma patients (n= 519) of the set of translation-related genes that were differentially spliced in squamous cell carcinomas (SCC[c]) compared to WT keratinocytes.
C. Ribosomal proteins exhibit particularly poor correlations between mRNA and protein levels. Histogram and density plot of the Spearman correlations between mRNA and protein levels in human head and neck squamous cell carcinoma patients [25].
D. Correlation of *RPL21* and *RPL29* mRNA and protein levels in human head and neck squamous cell carcinoma patients [25]. x- and y-axes are upper-quartile normalized expression of RNA and protein, respectively, as previously described [25].
E. Parallel coordinate plot for the TOP score and PRTE score of the same gene in WT and SCC light polysome (LP) and heavy polysome (HP) fractions. TOP scores are shown for genes with an average of more than 1. PRTE scores are shown for genes with an average of more than 10 PRTEs per million counts. Red line marks the median. P values indicate a Wilcoxon signed rank test.

Supplementary Fig. 3.
A-B. *RPL21* and *RPL29* top and bottom quartile mRNA expression analyses in all TCGA datasets suggesting that *RPL21* or *RPL29* mRNA expression significantly correlates with overall patient survival in different cancer types.

Supplementary Fig. 4.
A-B. 5′TOP motif-containing, but not TOP-less, *RPL21* transcripts correlate with shorter overall survival in human head and neck squamous cell carcinoma patients. According to the Ensembl annotation, four human *RPL21* transcripts encode identical proteins and only differ by their 5′UTR. Two transcripts contain a 5′TOP motif (red), one contains a TOP-like motif (blue, 5′ sequence shown below) and one transcript does not contain a 5′TOP motif. *RPL21* top and bottom quartile transcript isoform expression in human TCGA HNSCC patients′ samples (n=519).
